# HPV genotypes and associated cervical cytological abnormalities in women from the Pearl River Delta region of Guangdong province, China: a cross-sectional study

**DOI:** 10.1186/1471-2334-14-388

**Published:** 2014-07-12

**Authors:** Lipeng Jing, Xingming Zhong, Weihuang Huang, Yang Liu, Man Wang, Zhulin Miao, Xiaoping Zhang, Jing Zou, Baowen Zheng, Congde Chen, Xiaoman Liang, Guang Yang, Chunxia Jing, Xiangcai Wei

**Affiliations:** 1 Department of Epidemiology, Medical School of Jinan University, Jinan, Guangdong Province, China; 2 Family Planning Research Institute of Guangdong, Guangzhou, Guangdong, China; 3 Clinical Laboratory Center of the Beijing Genomics Institute (BGI), Shenzhen, Guangdong Province, China; 4 Kingmed Center for Clinical Laboratory, Guangzhou, Guangdong Province, China

**Keywords:** Human papillomavirus, Cytology, Cross-sectional study, Infection

## Abstract

**Background:**

It is important to understand the specific HPV genotype distribution in screen-detected lesions. HPV Genotype is helpful for separating HPV-positive women at greater risk of cancer from those who can regress spontaneously and for preventing cervical cancer at early stage. The aim of this study was to investigate the high-risk HPV genotype distribution among cervical cytology abnormality in Pearl River Delta Region, Southern China

**Methods:**

5585 HPV-infected women were screened from 77069 women in Pearl River Delta Region. Information was obtained from 3226 screened subjects through questionnaires and personal interviews. Exfoliated cervical cells were collected by doctors for HPV test with MassARRAY (Sequenom, Sandiego, CA) technique based on the matrix-assisted laser desorption/ionization time-of flight (MALDI-TOF) mass spectrometry (MS). The ThinPrep cytology test was performed to screen for cervical cancer. Unconditional logistic was used to determine the most common HPV carcinogenic types.

**Results:**

Of the 3226 HPV-positive samples tested, 1744 (54.1%) with normal cervical cytology, 1482 (45.9%) with abnormal cytology. The five most common HPV types in this study were HPV16 (20.2%), HPV52 (17.1%), HPV58 (13.2%), HPV18 (9.5%), HPV6 (7.6%). Overall, HPV16 (OR = 10.5, 95% CI: 3.7 ~ 29.6), HPV33 (OR = 9.1, 95% CI: 2.8 ~ 29.2), HPV58 (OR = 6.3, 95% CI: 2.1 ~ 18.6), HPV31 (OR = 4.5, 95% CI: 1.3 ~ 15.5), multiple genotype infection (OR = 3.0, 95% CI: 1.7 ~ 14.7), especially HPV16 and HPV33, increased the risk of cytology abnormalities.

**Conclusions:**

HPV16, HPV31, HPV33, HPV58, and multiple HPV genotype infection increased the risk of cytology abnormalities in Pearl River Delta Region and might be useful for the screening, preventing, treating, and monitoring of pre-cancer lesions in southern China.

## Background

Cervical cancer is estimated to be the one of the leading causes of cancer among women worldwide; approximately 530000 new cases and 275000 resultant deaths are reported each year. About 85% of cervical cancer cases occur in developing countries [1]. Infection with certain high-risk human papillomavirus (HPV) types has a causal and necessary role in the development of cervical cancer and its precursor lesions [2,3]. More than 150 HPV types have been identified [4], of the 30 high risk (HR) HPV types, 15 are carcinogenic and infect the genitourinary mucosa, which is highly associated with the development of cervical cancer [5,6]. HPV16 is the most carcinogenic type by far, followed by HPV18, 58, 33, 52, 45, 31, and 35; together, these account for about 90% of cervical cancer cases in Asia [7,8]. The low risk (LR) HPV types HPV6 and 11 are associated with low-grade cervical lesions and about 90% of genital warts [9].

Besides HPV16 and HPV18, the next most common types in all world regions, other high-risk carcinogenic types, namely HPV31, HPV33, HPV45, HPV52, HPV58 and HPV35 have been reported with different distribution across world regions. Sub- Saharan Africa with equally HPV35 prevalence with HPV16, and the second most common types were HPV31 in Europe, HPV58 in South America, and HPV33 in Asia. Heterogeneity of HPV infection was significant across Asia, especially for HPV58 [10].

Notably, even high-risk HPV genotypes can contribute to different progression of cervical disease [11]. A meta-analysis from the IARC highlights the importance of HPV type in the risk of progression to cancer, even from HSIL by comparing the HPV type-distribution across cervical lesions [12]. Furthermore, HPV type-distribution has significant shifts across cervical lesions of increasing severity [13], which indicate that there is great potential use of HPV genotype not only in improving risk stratification of women with HPV infection in cervical screening programmes, but also in separating HPV-positive women at greater risk of cancer from those who can regress spontaneously.

There were about 58000 new cervical cases and approximately 20000 deaths in China [14]. And the widely variety of the incidence and mortality of cervical cancer among different population, geographic area and time period in China complicate the potential HPV genotype attribution to cervical disease [14].

The Pearl River Delta Region (PRD) is located in mid-southern coastal part of Guangdong, Southern China, which with a total area over 56000 km^2^ and with a population over 47 million. Notably, this area accounts for 10% of China’s gross domestic product (GDP) and over 80% of Guangdong’s GDP, which shows highly concentration of economy activity. Genotyping can be helpful for estimating the attribution of HPV genotype in this area [15]. Thus, it is important to understand the specific HPV genotype distribution in screen-detected lesions which is helpful for preventing women with high risk of cervical disease. The aim of this study was to investigate the HPV genotype distribution among cervical cytology abnormality in PRD and to determine the most prevalent genotype in cervical cytology disease grades.

## Methods

### Participants

**Figure 1:**
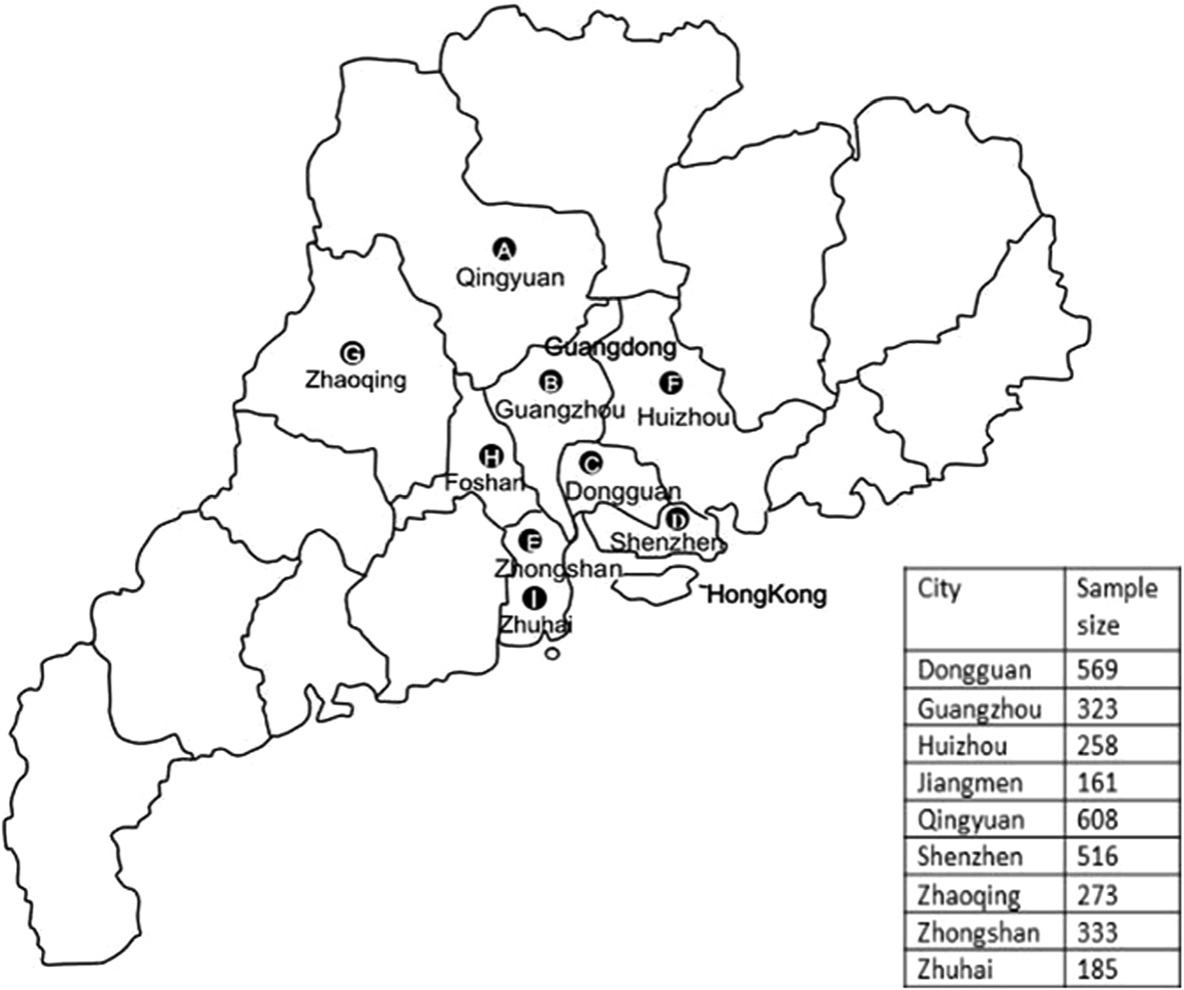
**Nine studied cities (A–I) in the Pearl River Delta region of Guangzhou, China.** Geographic locations of the nine cities (A–I) in the Pearl River Delta region of Guangzhou, China, represented by the study population.

The study was conducted between May 2011 and November 2012 in Guangdong province, China. 5585 women were screened from 77069 women from nine cities, of these 2359 were excluded. Subjects were women between the ages of 18 and 68 years old (median age: 38.0 years) from nine cities in the Pearl River Delta region: Dongguan, Guangzhou, Huizhou, Jiangmen, Qingyuan, Shenzhen, Zhaoqing, Zhongshan, and Zhuhai (Figure 1). Eligible women were sexually active, had an intact uterus, not pregnant, and had no history of miscarriage, cervical cancer, medical treatment, or surgery. Most women in the study had never been screened for cervical cancer within the three preceding years. All eligible women came to the Family Planning Service Stations or nearby medical institutions of each city, where the study was explained to each patient and written consent for their participation was obtained. Then the information and exfoliated cervical cells were collected by doctors. The study protocol was approved by the research ethics committee of the Family Planning Research Institute of Guangdong province.

### HPV testing

Exfoliated cervical cell samples were collected from vaginal swabs and conserved in 2.5 ml denaturation buffer (Qiagen, Valencia). Total DNA from cervical cells was extracted using the commercial magnetic beads kit (Chemagen, Pekinelmer, Waltham, MA) according to the manufacturer’s instructions. Then 16 HPV genotypes were detected, including HPV16, HPV18, HPV31, HPV33, HPV35, HPV39, HPV45, HPV51, HPV52, HPV56, HPV58, HPV59, HPV66, and HPV68 (all HR HPV); and HPV6 and HPV11 (both LR HPV) with MassARRAY (Sequenom, Sandiego, CA) technique based on the matrix-assisted laser desorption/ionization time-of flight (MALDI-TOF) mass spectrometry (MS) [16,17]. All these procedures were performed in the clinical standard laboratory of BGI (Beijing Genomics Institute, Shenzhen, China).

### ThinPrep cytological test

Another cervical exfoliated cells sample was collected by cyto-brush (QIAGEN, Valencia, CA) among women with HPV infection. ThinPrep 2000 (Hologic Inc.) and SurePath liquid-based Pap test (BD, USA) were used for the TCT test which was performed by Kingmed Center for Clinical Laboratory Corporation (Guangzhou, China). Cytological slides were read by three cytopathologist. Cervical cell samples were categorized according to the Bethesda System (2001) [18,19] as follows: normal; atypical squamous cells of unknown significance (ASCUS); low-grade squamous intraepithelial neoplasia (LSIL); and high-grade squamous intraepithelial lesion (HSIL) or worse (i.e., cases of squamous cell carcinoma were combined with HSIL cases for the purposes of analysis).

### Statistical analysis

The *χ*^2^ test was used to compare the differences in HPV genotype representation for different cytological grades. Independent predictors for cervical lesions across different HPV genotype infection were evaluated via unconditional logistic regression with odds ratio (ORs) and 95% confidence intervals (CIs) (LR-HPV: HPV6 or HPV11 as reference). Data analysis was performed using SPSS13.0 (SPSS, Chicago, USA). All statistical tests were two-sided; P values < 0.05 were considered statistically significant.

## Results

### Population characteristic

**Figure 2:**
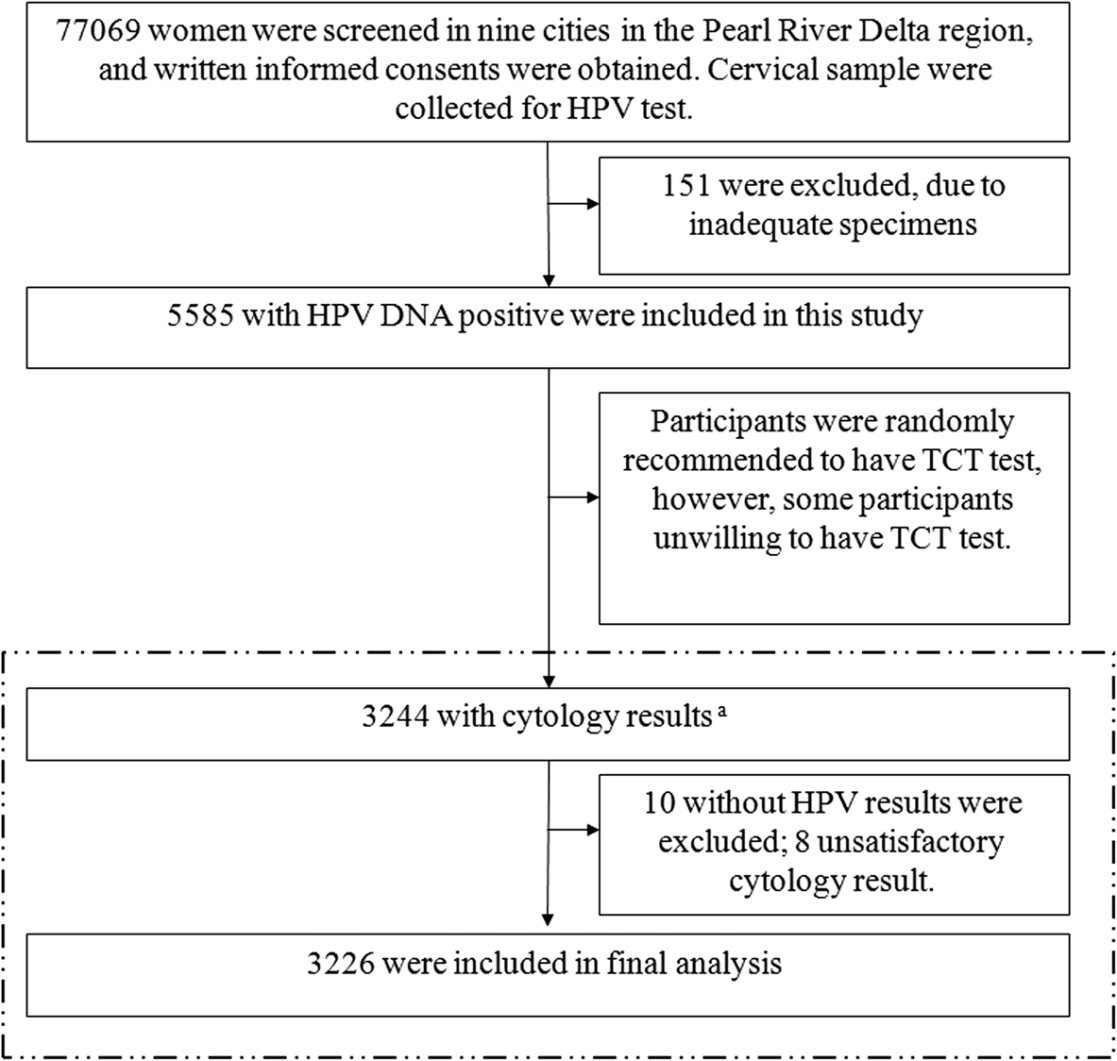
**Flow chart of study participants. **^a^All women were recommended to underwent further treatment according to <2013 ASCCP Guidelines on Cervical Cancer Screening and Management in US> . our study was shown with internal the dotted-line frame. HPV = human papillomavirus. TCT = Thinprep cytologic test.

The study participants came from nine cities in Guangdong’s PRD, China, where is adjacent to Hong Kong, one of the most prosperous city in China (Figure 1). Figure 2 shows the flow of study participants. Of the 3226 HPV-positive women included in the analysis, the median age was 38.0 years, almost all of the women reported 0-1 sexual partners and the mean Years of sexual active life was 14.4 years, women aged 30-34 years accounted for the largest proportion (67.0%) of participants. Only 490 (15.5%) women had attended college. A summary of participants’ characteristics was shown in Table 1. Among 3226 women, 1744 cases (54.1%) was normal cervical cytology, 632 cases (19.6%) with ASCUS, 237 cases (7.3%) with LSIL, and 237 cases (7.3%) with HSIL (Table 2).

**Table 1:** The demographics of participants

**Characteristics**	**No. of cases**^**a**^	**Percentage (%)**
Age (years)		
<30	480	14.9
30~	2158	67.0
45~	585	18.2
Years of sexual active life		
<15	1692	52.4
> = 15	1534	47.6
Number of sexual partners		
0-1	2853	97.7
2-3	64	2.2
>3	3	0.1
Educational level		
Elementary school and below (%)	350	9.6
Junior middle school (%)	1368	43.1
High school (%)	1008	31.8
College and above (%)	490	15.5

^a^Numbers did not always sum to the total due to missing data.

**Table 2:** Cytology findings founded in 3226 women studied

**Cytology grade**^**a**^	**n**	**%**
Normal	1744	54.1
ASCUS	632	19.6
LSIL	613	19.0
HSIL	237	7.3
Total	3226	100.0

^a^ASCUS, atypical squamous cells of undetermined significance; LSIL, low-grade squamous intraepithelial lesions; HSIL, high-grade squamous intraepithelial lesion.

### HPV genotypes and cytology grade

**Figure 3:**
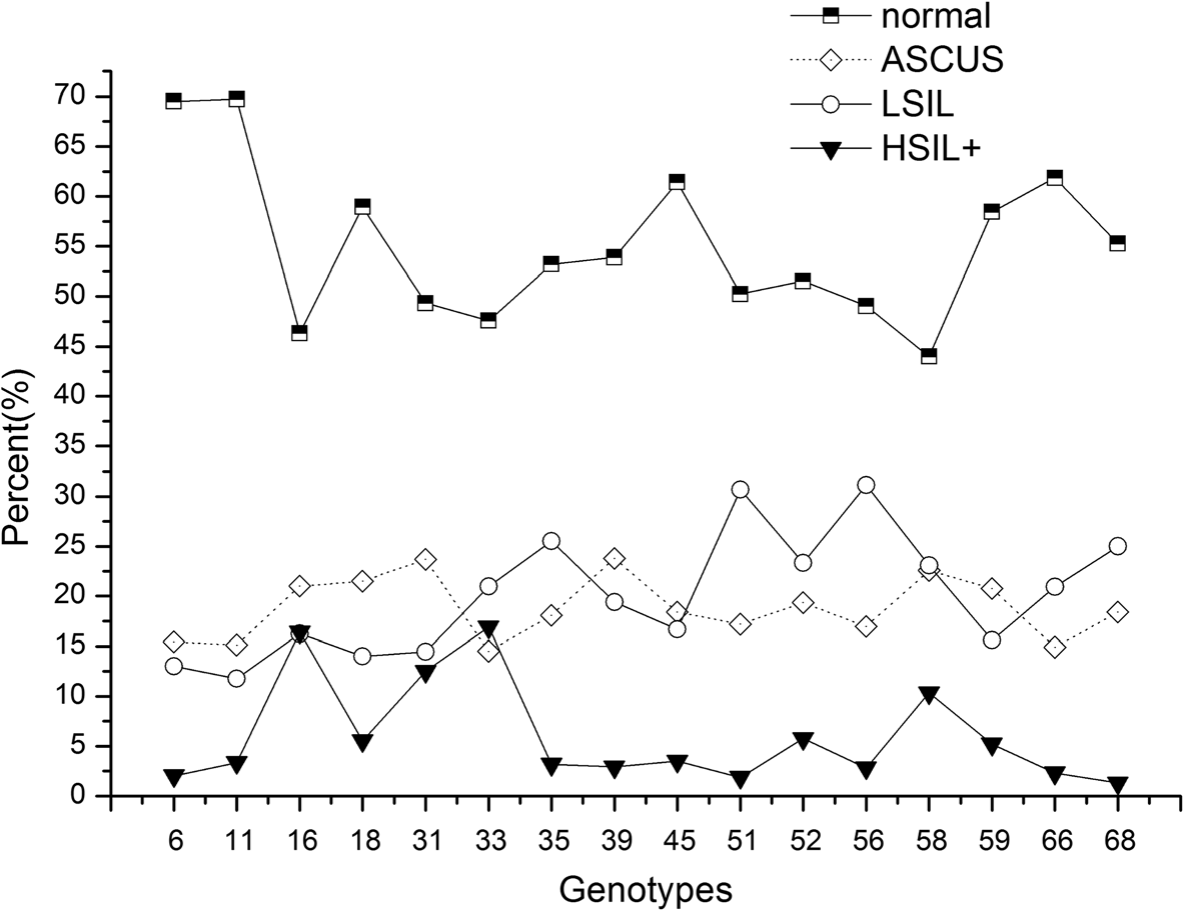
**Distribution of HPV genotypes among 3226 women with HPV-positive cervical exfoliated cell.** The cervical exfoliated cell had a cytology classification of normal (n = 1744), ASCUS (n = 632), LSIL (n = 613), or HSIL + (n = 237).

The most common HPV genotypes were HPV16 (20.2%), followed by HPV52 (17.1%), HPV58 (13.2%), HPV18 (9.5%), HPV6 (7.6%), HPV45 (7.1%), HPV51 (6.7%), HPV66 (6.7%), and HPV39 (6.4%). Across all cytology grades, HPV16 was the most prevalent type than that of any other HR-HPV genotype. HPV6, HPV11, HPV45, and HPV66 were prone to be concentrated in normal cytology than HSIL. Similar percentages were detected for each HPV type between ASCUS and LSIL except for HPV51 and HPV56 in LSIL (Table 3, Figure 3).

**Table 3:** Distribution of HPV genotypes among women with different cytology grades

	**Normal**		**ASCUS**		**LSIL**		**HSIL+**	
	**n**	**%**	**n**	**%**	**n**	**%**	**n**	**%**
HPV6	171	69.5	38	15.5	32	13.0	5	2.0
HPV11	83	69.8	18	15.1	14	11.8	4	3.4
HPV16	302	46.3	137	21.0	106	16.3	107	16.4
HPV18	181	59.0	66	21.5	43	14.0	17	5.5
HPV31	75	49.3	36	23.7	22	14.5	19	12.5
HPV33	59	47.6	18	14.5	26	21.0	21	16.9
HPV35	50	53.2	17	18.1	24	25.5	3	3.2
HPV39	111	53.9	49	23.8	40	19.4	6	2.9
HPV45	140	61.4	42	18.4	38	16.7	8	3.5
HPV51	108	50.2	37	17.2	66	30.7	4	1.9
HPV52	285	51.5	107	19.4	129	23.3	32	5.8
HPV56	52	49.1	18	17.0	33	31.1	3	2.8
HPV58	187	44.0	96	22.6	98	23.1	44	10.4
HPV59	45	58.4	16	20.8	12	15.6	4	5.2
HPV66	133	61.9	32	14.9	45	20.9	5	2.3
HPV68	42	55.3	14	18.4	19	25.0	1	1.3

### The risk factors of HSIL+

**Figure 4:**
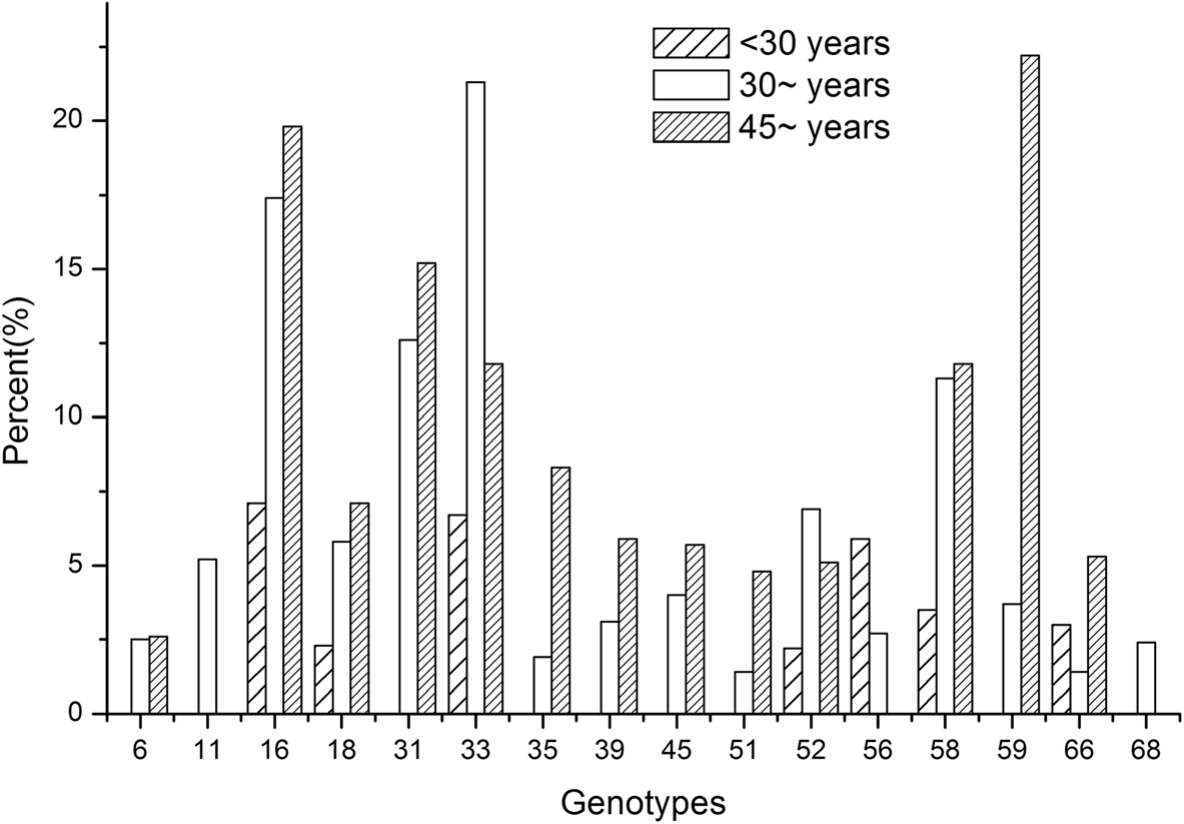
**Distribution of HPV genotypes among women with HSIL + cytology stratified by age.** Of the 237 women diagnosed HSIL, the percentage of HPV16, HPV31, and HPV59 were significantly higher among women age above 45 years old than women less than 45 years old, but there was no HPV11, HPV56, or HPV68 infection among women with HSIL whom aged above 45 years old. HPV33 were more common in women aged 30 ~ 44 years old than other age groups. HPV58 were more common in women aged 30 or older than women aged less than 30 years.

In multivariable analyses, women with more than 15 years active sexual life were more likely to be HSIL + compared to less than 5 years (OR = 3.9, P < 0.001, 95% CI: 1.9 ~ 8.0) (Table 4). Women with high school education were more likely to be HSIL + compared to elementary school and below (OR = 2.4, P < 0.01, 95% CI: 1.4 ~ 4.3). Detection of HSIL + was more common among women with over 4 times of birth than women with 0-1 time birth (OR = 3.6, P < 0.001, 95% CI: 1.7 ~ 7.8). As we can see from Figure 3, HPV16, HPV33, HPV58, and HPV31 showed high percentage in HSIL + but low percentage in normal. Overall, HPV16, HPV58 and HPV33 (especially HPV16 and HPV33) increased the risk of HSIL + (Figure 3). Notably, as shown in Table 4, detection of HSIL + was significantly more common among women with HPV16 (OR = 10.5, P < 0.001, 95% CI: 3.7 ~ 29.6), HPV33 (OR = 9.1, P < 0.001, 95% CI: 2.8 ~ 29.2), HPV58 (OR = 6.315, P < 0.001, 95% CI: 2.1 ~ 18.6), HPV31 (OR = 4.5, P < 0.05, 95% CI: 1.3 ~ 15.5), multiple genotype infection (OR = 3.0, P < 0.01, 95% CI: 1.7 ~ 14.7) compared with women only with HPV6 or HPV11. Although the proportion of HPV52 infection were higher in HSIL, there was no significant difference between cytology grades was observed (HPV52 vs. LR-HPV: OR = 2.9, P > 0.05, 95% CI 0.9 ~ 8.8) (data were not shown).Furthermore, of the 237 women with HSIL, the percentage of HPV16, HPV31, and HPV59 were significantly higher among women age above 45 years old than women less than 45 years old, but there was no HPV11, HPV56, or HPV68 infection among women with HSIL whom aged above 45 years old (Figure 4). HPV33 were more common in women aged 30 ~ 44 years old than other age groups. HPV58 were more common in women aged 30 or older than women aged less than 30 years.

**Table 4:** Multivariate analyse of determinants for cytology grades (HSIL+) among women with human papillomavirus infection from PDR

***Characteristic***	***No. of women with HSIL or Normal detected***	***OR***	***P***	***95% CI***
HPV types				
only LR-HPV	125	ref.	ref.	ref.
HPV16	288	10.49	<0.01	3.72 ~ 29.62^**^
HPV31	65	4.53	<0.05	1.32 ~ 15.52^*^
HPV33	60	9.06	<0.01	2.81 ~ 29.21^**^
HPV58	167	6.32	<0.01	2.14 ~ 18.60^**^
Multiple genotype infection	206	4.99	<0.01	1.70 ~ 14.71^**^
Years of sexual active life				
0~	233	ref.	ref.	ref.
5~	261	1.89	0.14	0.81 ~ 4.39
10~	423	2.87	<0.05	1.36 ~ 6.08^*^
15~	788	3.87	<0.01	1.87 ~ 8.03^**^
Education level				
Elementary school and below	168	ref.	ref.	ref.
Junior middle school	723	1.32	0.35	0.74 ~ 2.34
High school	559	2.43	<0.01	1.35 ~ 4.35^**^
College and above	255	1.31	0.47	0.63 ~ 2.73
Birth times				
0-1	943	ref.	ref.	ref.
2-3	719	1.38	<0.05	0.97 ~ 1.96^*^
4+	43	3.62	<0.01	1.68 ~ 7.83^**^

Note. Totals may be less than stated sample size due to missing data. The multivariable model included data on 1705 women due to missing data on correlates. HPV = Human papillomavirus, LR-HPV = low-risk human papillomavirus, OR = odds ratio, CI: confidence interval, ref. =referent group. ^*^P < 0.05, ^**^P < 0.01.

## Discussion

It is necessary to know the predominant HPV genotypes associated with cervical lesions with the aim of preventing cervical cancer. Our study presented the distribution of HPV genotypes and the association with cytological characteristics in a population consisted of 3226 women with HPV positive and an age range between 18 and 68 years in PDR, Southern China.

Findings from our analysis show that HPV16 was the most predominant genotype in HPV positive women in PRD, which is similar with the most part of the world. Besides HPV16, HPV52 and HPV58 were also common in this study. These results are consistent with studies confined within part of the same geographical area [19,20]. Also, HR-HPV genotypes with relatively high proportion in our study (i.e., HPV16, and HPV58) were consistent with the study in Asia but different with Europe, Latin America and Caribbean, Africa, and Oceania [21]. Notably, percentage of HPV16, HPV31, HPV33, and HPV58 was significantly higher in women with HSIL or worse group than other HPV types (Table 3), which is consistent with the Hou R’s study [22]. All these four HPV types were characterized as high-risk and carcinogenic HPV types [3], which were not only with high prevalence in the population [20], but also with long duration time [23-25]. We also found that multiple HPV genotype infections increased the risk of HSIL compared with LR-HPV in our study, but it cannot distinguish low-grade from high-grade cytological lesions [26], thus, we speculated that infection with specific HPV genotypes (i.e., HPV16, HPV31, HPV33, and HPV58) may more likely heightened risks for progression of cervical dysplasia and carcinoma in the studied population.

Previous studies showed that HPV52 was one of the predominant genotypes in our study region and also in other parts of China [19,27,28], but it does not increase the risk of HSIL in our study (the proportion of HPV33 and HPV58 in HSIL in the present study was significantly higher than that of HPV52 which the proportion was similar to HPV59), which might result from the method we used, geographic factors, or the polymorphism of HPV52 contributed to the underestimation of it [29].

The distribution of HPV genotype and risk of cytology abnormalities in the present study are different from those in previous studies, which may be the variations in the study design, specimen types, screening methods [30]. Our study did not support that the risk of HSIL + was different in various age groups among women with HPV infection. But we stratified by age to reduce confounding and were able to describe the distribution of HPV genotypes among women with different cytology grades in various age groups (Additional file 1: Table S1). In our findings, there is a discrepancy of the HPV genotype distribution assigned different age groups. Women less than 30 years old are more likely infected by HPV52, followed by HPV16, HPV58, and HPV6. Women aged 30-44years old are more likely infected by HPV16, HPV52, and women more than 45 years old are more prone to be infected by HPV58. Interestingly, HPV31 which may increase the risk of cytology disease were more likely concentrated in normal compared with HSIL group less than 30 years old (Additional file 1: Table S1) which revealed that different aged women harbour specific HPV genotype might with different progressive progression. Furthermore, women with HPV infection in different age have different potential of progression [31]. So, it is of great importance of clarifying the potential role of specific HPV genotype infection in carcinogenesis to evaluate the risk and contribution of carcinogenesis among different aged population in the future. Our data can move forward the estimate of genotype attribution and gain a better understanding of the potential role of the prevalent genotypes in studied region.

Furthermore, determining the causal attribution of carcinogenic HPV genotypes to cervical disease is important in the application of HPV genotypes included in future screening assays, and in the management and developing prophylactic vaccines. HPV-based primary screening is not only more sensitive for earlier detection of clinically high-grade cervical lesions than cytology [32], but also is suit for carrying out in lower-resource countries like China [33]. Early detection of clinically high-grade cervical lesions caused by specific HPV types may was a major component of the benefit. However, HPV-based screening is more inclined to the detection of lesions with lower potential for progression and at an earlier age [25,34,35], thus potentially affecting quality of life. Even high-risk HPV genotypes can contribute to different progression of cervical disease [11].

The design of our study should be considered when interpreting our results. Our data are from a cross-sectional study which was designed to identify detriments of early-stage cervical disease, so all of the women were HPV-infected. Indeed, detection of cytology abnormalities was more common among the participants with HPV infection in our study compared to these samples randomly obtained from women in PRD. We do not believe, however, that study design solely accounts for the findings observed in our study. Although future study is needed to confirm the findings from this study, our data provide valuable initial insight into the distribution of HPV among PRD women and suggest that some specific HR-HPV may help explain the existing cervical cancer disparities in PRD, furthermore, our results not only give support to the use of HPV testing, especially including HPV16, HPV58, HPV31, and HPV33 testing for women aged 30 years and older but also provided valuable fundamental data to the future study with the aim of reducing the burden of cervical cancer, last but not least, these results give us a hint that may there is great potential use of these HPV genotypes in improving risk stratification of women with HPV infection in cervical screening programmes, and in improving performance and cost-effectiveness of programs while reducing over treatment and patient anxiety.

There are some limitations in our study. Since we analyzed a population based on HPV-positive results from a large cross-sectional study in Guangdong, women without HPV infection that cause cytological abnormalities are underrepresented in our population. Furthermore, the accuracy of the information about their sexual and reproductive history might impact the authenticity of this survey.

In summary, the results of this study provide clear indication of the HPV infections among cytology abnormal women in the Pearl River Delta region of Guangdong Province of China, and only a few high-risk HPV genotypes might be more useful for the screening and monitoring of pre-cancer lesions.

## Conclusions

HPV16, HPV31, HPV33, HPV58, and multiple HPV genotype infection increased the risk of cytology abnormalities in Pearl River Delta Region and might be useful for the screening, preventing, treating, and monitoring of pre-cancer lesions in southern China.

## Competing interests

All authors declare that they have no competing interests.

## Authors’ contributions

LPJ and XMZ wrote the manuscript. XCW, GY and CXJ conceived the study, assisted in drafting the article for important intellectual content. WHH, YL and MW performed the surveys and analyzed the data collected. ZLM, XPZ and JZ performed the HPV test and analyzed the results. BWZ, CDC and XML performed the cytology study and interpreted the results. All authors participated to the design of the study. All authors read and approved the final manuscript.

## Pre-publication history

The pre-publication history for this paper can be accessed here:

http://www.biomedcentral.com/1471-2334/14/388/prepub

## Supplementary Material

Additional file 1: Table S1 Distribution of HPV genotypes among different cytology grades stratified by age^a^. Click here for file
