# Supplementary material for: HPV genotypes and associated cervical cytological abnormalities in women from the Pearl River Delta region of Guangdong province, China: a cross-sectional study
Source: BMC Infect Dis. 2014 Jul 12;14:388. doi: 10.1186/1471-2334-14-388 (PMC4226991; doi:10.1186/1471-2334-14-388)
Supplement: Additional file 1: Table S1 — Distribution of HPV genotypes among different cytology grades stratified by agea. [file 1471-2334-14-388-S1.doc]

# Additional files

### Additional file 1: Table S1–Distribution of HPV genotypes among different cytology grades stratified by age ^a^

| HPV  genotype | Age  (years) | Normal  n (%) | ASCUS  n (%) | LSIL  n (%) | HSIL or worse  n (%) | Total number of women |
| --- | --- | --- | --- | --- | --- | --- |
| HPV6 | <30 | 32(69.6) | 5(10.9) | 9(19.6) | 0(0.0) | 46 |
|  | 30~ | 111(68.9) | 26(16.1) | 20(12.4) | 4(2.5) | 161 |
|  | 45~ | 28(73.7) | 6(15.8) | 3(7.9) | 1(2.6) | 38 |
| HPV 11 | <30 | 21(72.4) | 4(13.8) | 4(13.8) | 0(0.0) | 29 |
|  | 30~ | 53(68.8) | 10(13.0) | 10(13.0) | 4(5.2) | 77 |
|  | 45~ | 9(69.2) | 4(30.8) | 0(0.0) | 0(0.0) | 13 |
| HPV 16 | <30 | 47(55.3) | 17(20.0) | 15(17.6) | 6(7.1) | 85 |
|  | 30~ | 208(44.7) | 99(21.3) | 77(16.6) | 81(17.4) | 465 |
|  | 45~ | 46(45.5) | 21(20.8) | 14(13.9) | 20(19.8) | 101 |
| HPV 18 | <30 | 29(65.9) | 7(15.9) | 7(15.9) | 1(2.3) | 44 |
|  | 30~ | 118(57.0) | 49(23.7) | 28(13.5) | 12(5.8) | 207 |
|  | 45~ | 34(60.7) | 10(17.9) | 8(14.3) | 4(7.1) | 56 |
| HPV 31 | <30 | 7(87.5) | 1(12.5) | 0(0.0) | 0(0.0) | 8 |
|  | 30~ | 56(50.5) | 26(23.4) | 15(13.5) | 14(12.6) | 111 |
|  | 45~ | 12(36.4) | 9(27.3) | 7(21.2) | 5(15.2) | 33 |
| HPV 33 | <30 | 8(53.3) | 2(13.3) | 4(26.7) | 1(6.7) | 15 |
|  | 30~ | 35(46.7) | 10(13.3) | 14(18.7) | 16(21.3) | 75 |
|  | 45~ | 16(47.1) | 6(17.6) | 8(23.5) | 4(11.8) | 34 |
| HPV 35 | <30 | 10(55.6) | 4(22.2) | 4(22.2) | 0(0.0) | 18 |
|  | 30~ | 27(51.9) | 9(17.3) | 15(28.8) | 1(1.9) | 52 |
|  | 45~ | 13(54.2) | 4(16.7) | 5(20.8) | 2(8.3) | 24 |
| HPV 39 | <30 | 25(61.0) | 8(19.5) | 8(19.5) | 0(0.0) | 41 |
|  | 30~ | 69(52.7) | 31(23.7) | 27(20.6) | 4(3.1) | 131 |
|  | 45~ | 17(50.0) | 10(29.4) | 5(14.7) | 2(5.9) | 34 |
| HPV 45 | <30 | 34(79.1) | 6(14.0) | 3(7.0) | 0(0.0) | 43 |
|  | 30~ | 84(56.0) | 31(20.7) | 29(19.3) | 6(4.0) | 150 |
|  | 45~ | 22(62.9) | 5(14.3) | 6(17.1) | 2(5.7) | 35 |
| HPV 51 | <30 | 13(48.1) | 6(22.2) | 8(29.6) | 0(0.0) | 27 |
|  | 30~ | 70(47.9) | 28(19.2) | 46(31.5) | 2(1.4) | 146 |
|  | 45~ | 25(59.5) | 3(7.1) | 12(28.6) | 2(4.8) | 42 |
| HPV 52 | <30 | 52(58.4) | 13(14.6) | 22(24.7) | 2(2.2) | 89 |
|  | 30~ | 181(49.7) | 73(20.1) | 85(23.4) | 25(6.9) | 364 |
|  | 45~ | 51(51.5) | 21(21.2) | 22(22.2) | 5(5.1) | 99 |
| HPV 56 | <30 | 10(58.8) | 4(23.5) | 2(11.8) | 1(5.9) | 17 |
|  | 30~ | 35(46.7) | 9(12.0) | 29(38.7) | 2(2.7) | 75 |
|  | 45~ | 7(50.0) | 5(35.7) | 2(14.3) | 0(0.0) | 14 |
| HPV 58 | <30 | 28(49.1) | 15(26.3) | 12(21.1) | 2(3.5) | 57 |
|  | 30~ | 108(40.6) | 59(22.2) | 69(25.9) | 30(11.3) | 266 |
|  | 45~ | 51(50.0) | 22(21.6) | 17(16.7) | 12(11.8) | 102 |
| HPV 59 | <30 | 6(42.9) | 4(28.6) | 4(28.6) | 0(0.0) | 14 |
|  | 30~ | 33(61.1) | 12(22.2) | 7(13.0) | 2(3.7) | 54 |
|  | 45~ | 6(66.7) | 0(0.0) | 1(11.1) | 2(22.2) | 9 |
| HPV 66 | <30 | 22(66.7) | 3(9.1) | 7(21.2) | 1(3.0) | 33 |
|  | 30~ | 89(61.8) | 23(16.0) | 30(20.8) | 2(1.4) | 144 |
|  | 45~ | 22(57.9) | 6(15.8) | 8(21.1) | 2(5.3) | 38 |
| HPV 68 | <30 | 9(56.2) | 4(25.0) | 3(18.8) | 0(0.0) | 16 |
|  | 30~ | 25(59.5) | 5(11.9) | 11(26.2) | 1(2.4) | 42 |
|  | 45~ | 8(44.4) | 5(27.8) | 5(27.8) | 0(0.0) | 18 |

^a^ values are given as number (percentage). HPV, human papillomavirus.

ASCUS, atypical squamous cells of undetermined significance; LSIL, low-grade squamous intraepithelial lesions; HSIL, high-grade squamous intraepithelial lesion.
